# Value of screening and follow‐up brain MRI scans in patients with metastatic melanoma

**DOI:** 10.1002/cam4.4342

**Published:** 2021-11-05

**Authors:** Annemarie C. Eggen, Thijs T. Wind, Ingeborg Bosma, Miranda C. A. Kramer, Peter Jan van Laar, Hiska L. van der Weide, Geke A. P. Hospers, Mathilde Jalving

**Affiliations:** ^1^ Department of Medical Oncology University of Groningen University Medical Center Groningen Groningen The Netherlands; ^2^ Department of Neurology University of Groningen University Medical Center Groningen Groningen The Netherlands; ^3^ Department of Radiation Oncology University of Groningen University Medical Center Groningen Groningen The Netherlands; ^4^ Department of Radiology Ziekenhuisgroep Twente, Almelo, and Hengelo Almelo The Netherlands; ^5^ Department of Radiology University of Groningen University Medical Center Groningen Groningen The Netherlands

**Keywords:** brain metastases, early diagnosis, melanoma, neuroimaging, neuro‐oncology

## Abstract

**Background:**

Novel treatments make long‐term survival possible for subsets of patients with melanoma brain metastases. Brain magnetic resonance imaging (MRI) may aid in early detection of brain metastases and inform treatment decisions. This study aimed to determine the impact of screening MRI scans in patients with metastatic melanoma and follow‐up MRI scans in patients with melanoma brain metastases.

**Methods:**

This retrospective cohort study included patients diagnosed with metastatic melanoma or melanoma brain metastases between June 2015 and January 2018. The impact of screening MRI scans was evaluated in the first 2 years after metastatic melanoma diagnosis. The impact of follow‐up MRI scans was examined in the first year after brain metastases diagnosis. The number of MRI scans, scan indications, scan outcomes, and changes in treatment strategy were analyzed.

**Results:**

In total, 116 patients had no brain metastases at the time of the metastatic melanoma diagnosis. Twenty‐eight of these patients (24%) were subsequently diagnosed with brain metastases. Screening MRI scans detected the brain metastases in 11/28 patients (39%), of which 8 were asymptomatic at diagnosis. In the 96 patients with melanoma brain metastases, treatment strategy changed after 75/168 follow‐up MRI scans (45%). In patients treated with immune checkpoint inhibitors, the number of treatment changes after follow‐up MRI scans was lower when patients had been treated longer.

**Conclusion(s):**

Screening MRI scans aid in early detection of melanoma brain metastases, and follow‐up MRI scans inform treatment strategy. In patients with brain metastases responding to immune checkpoint inhibitors, treatment changes were less frequently observed after follow‐up MRI scans. These results can inform the development of brain imaging protocols for patients with immune checkpoint inhibitor sensitive tumors.

## Graphical abstract

This study demonstrates that screening magnetic resonance imaging (MRI) scans aid in the early detection of asymptomatic brain metastases. Furthermore, changes in treatment strategy often occur after follow‐up MRI scans in patients with melanoma brain metastases.

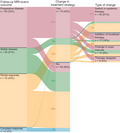

## 1 INTRODUCTION

Melanoma has the highest propensity of all cancers to metastasize to the brain, with up to 50% of the patients with metastatic melanoma developing brain metastases.^1^, ^2^, ^3^, ^4^ Patients with brain metastases frequently suffer from neurological sequelae and have shorter survival than patients without brain metastases.^5^ In the last decade, the melanoma treatment landscape has changed dramatically following the introduction of effective systemic treatments, including immune checkpoint inhibitors and BRAF/MEK inhibitors, and the increased use of stereotactic radiotherapy (SRT).^6^, ^7^, ^8^, ^9^, ^10^, ^11^, ^12^, ^13^, ^14^ The greatly improved survival and the chance of long‐term disease control have resulted in increased utilization of imaging modalities. However, evidence for optimal use of imaging modalities in the management of metastatic melanoma is lacking.

Treatment with immune checkpoint inhibitors can result in long‐term intracranial and extracranial responses.^6^, ^7^, ^13^ Response rates to combinational immune checkpoint inhibitors (nivolumab plus ipilimumab) range from 46% to 57% in patients with asymptomatic melanoma brain metastases.^6^, ^7^, ^9^ In contrast, intracranial response rates in patients with symptomatic brain metastases and/or leptomeningeal metastases are lower (5%–22%).^6^, ^7^, ^8^, ^9^ In patients with *BRAF*‐mutated melanoma, treatment with BRAF/MEK inhibitors demonstrate intracranial response rates of 44%–68%.^11^, ^13^, ^14^ However, the median duration of intracranial response is limited (4–6 months).^11^, ^13^, ^14^ In contrast to immune checkpoint inhibitor response rates, intracranial response rates to BRAF/MEK inhibitors are independent of symptoms.^10^, ^11^ For the localized treatment of melanoma brain metastases, SRT is most often the preferred choice of treatment. The treatment outcomes of SRT, including tumor response and treatment toxicity, such as radionecrosis, are better in patients with small volume brain metastases.^15^, ^16^, ^17^, ^18^, ^19^

Small volume and asymptomatic brain metastases are, thus, associated with better treatment outcomes, making early diagnosis of asymptomatic brain metastases and intracranial progression essential.^13^, ^16^, ^18^ Brain magnetic resonance imaging (MRI) scan is considered the gold standard for the diagnosis and follow‐up of brain metastases.^20^, ^21^, ^22^ For patients with metastatic melanoma, the National Comprehensive Cancer Network (NCCN; 2021)^23^ and European Society of Medical Oncology (2019)^24^, ^25^ guidelines recommend brain MRI scans at the time of staging. Additionally, the NCCN guideline also recommends MRI scans in patients with metastatic melanoma without brain metastases at the time of neurological symptoms and when the diagnosis of brain metastases would affect treatment decisions.^23^ However, information on the effectiveness of MRI scans as a screening tool to detect asymptomatic melanoma brain metastases at an early time point is scarce. When melanoma brain metastases are diagnosed, follow‐up brain MRI scans are advised to evaluate the treatment response every 2 or 3 months.^23^, ^24^, ^25^, ^26^, ^27^ How frequently these follow‐up MRI scans result in treatment strategy changes is yet to be determined.

Therefore, we aimed to determine the number of brain metastases being diagnosed asymptomatically by 6‐monthly screening MRI scans in patients with metastatic melanoma and the number of changes in treatment strategy after 3‐monthly follow‐up MRI scans in patients with melanoma brain metastases.

## 2 MATERIALS AND METHODS

This retrospective single‐center study consisted of patients diagnosed with metastatic melanoma and referred to the Department of Medical Oncology of the University Medical Center Groningen (UMCG), the Netherlands. Patients diagnosed with metastatic melanoma, without brain metastases at the time of diagnosis, between June 2015 and January 2018 were included to evaluate the impact of 6‐monthly screening MRI scans (Cohort 1). Patients diagnosed with melanoma brain metastases between June 2015 and January 2018 were included to evaluate the impact of 3‐monthly follow‐up MRI scans of melanoma brain metastases (Cohort 2). Patients included in the cohort that evaluated screening MRI scans (Cohort 1) could also be included in Cohort 2 if they developed brain metastases during follow‐up. Patients with follow‐up at other hospitals and patients that died from other invasive malignancies were excluded. Furthermore, due to the different biological behavior, different pattern of metastatic spread with much lower rate of brain metastases, and poor response rates to systemic treatment, we excluded patients diagnosed with ocular melanoma. The UMCG review board granted ethical approval and waived the need for an informed consent procedure (METc2017/511 and METc2019/361). The “opt‐out” register was assessed to exclude patients who disapprove of routinely collected data being used for research purposes.

### 2.1 Management of patients with melanoma in the UMCG

The UMCG is one of 14 certified melanoma centers in the Netherlands, and all standard of care treatments are available. According to clinical practice in the UMCG, patients diagnosed with metastatic melanoma received a brain MRI scan as part of standard diagnostic work‐up. If no brain metastases were present, the advise was to consider 6‐monthly brain MRI scans to screen for asymptomatic brain metastases. If brain metastases were diagnosed, 3‐monthly follow‐up MRI scans were advised to evaluate treatment response. Additional MRI scans were performed when clinically indicated. MRI scans were performed according to standardized imaging protocols, including at least T1 with and without contrast enhancement, T2, and FLAIR sequences. Patients with a contra‐indication for MRI scans had contrast‐enhanced CT scans instead. Very poor performance status, short life expectancy, or lack of treatment options were reasons to omit brain imaging. Besides regular brain MRI scans, the extracranial disease status was evaluated by regular CT scans, generally at 3‐month intervals.

### 2.2 Extraction of patient and tumor data

Age, gender, *BRAF*‐mutational status, LDH‐level at the time of diagnosis of metastatic melanoma and brain metastases, and the presence of neurological symptoms were extracted from electronic patient charts. Moreover, all anti‐tumor treatments received and changes in treatment strategy after MRI scans were documented. Changes in treatment strategy were defined as changes in systemic or localized anti‐tumor treatments (commencing, ceasing, or providing additional treatments). Additionally, shortening of the scan interval to less than 3 months was also considered a change in treatment strategy.

### 2.3 Evaluation of the impact of screening and follow‐up MRI scans

**FIGURE 1:**
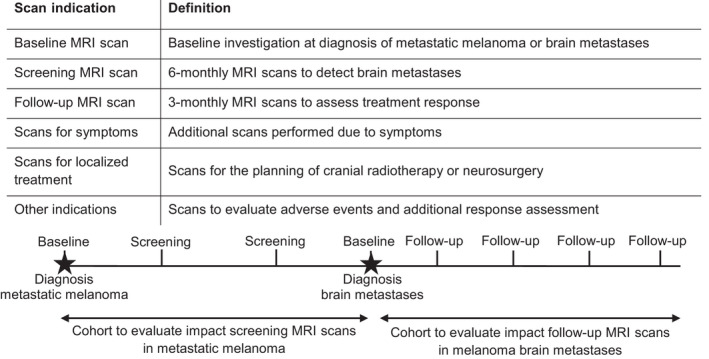
The different MRI indications and their definitions

The number of brain metastases diagnosed at an asymptomatic stage by screening MRI scans was assessed to determine the impact of 6‐monthly screening MRI scans. Therefore, the first 2 years after the metastatic melanoma diagnosis were retrospectively examined. The number of MRI scans and MRI indications were recorded. Figure 1 shows the different MRI indications and their definitions. Subsequently, scan outcomes were reviewed for the diagnosis of brain metastases. The presence of neurological symptoms before or within 1 week after the diagnosis of brain metastases was also documented. Patients who developed neurological symptoms within 1 week after brain metastases diagnosis were also considered to be diagnosed with symptomatic brain metastases. Furthermore, changes in treatment strategy (i.e., changes in anti‐tumor treatment or shortening of scan interval to less than 3 months) after brain metastases diagnosis were registered.

The number of changes in treatment strategy after follow‐up MRI scans was evaluated to determine the impact of 3‐monthly follow‐up MRI scans. First, all MRI scans performed within the first year after the diagnosis of brain metastases were identified including the scan indications. Subsequently, the outcomes of all follow‐up MRI scans were classified as progressive, stable disease, partial or complete response, according to the radiologist report. In mixed responses, the radiologist report, clinical patient notes, and scan images were used to classify the scan outcome. In cases of differing interpretations, the case was discussed until consensus was reached. Lastly, the changes in treatment strategy after follow‐up MRI scans were identified, and the influence of the MRI scans on treatment changes was classified by two melanoma Medical Oncologists (MJ and GAPH). For patients treated with BRAF/MEK inhibitors or immune checkpoint inhibitors, the number of treatment strategy changes after follow‐up MRI scans were also examined by time on treatment.

### 2.4 Statistical analyses

Statistical analyses were performed using SPSS Version 24.0 (IBM SPSS Statistics). Continuous variables were described using the mean and standard deviation or median and range, depending on data distribution. Kolmogorov–Smirnov tests, histograms, and Q–Q plots were used to analyze the data distribution. For categorical variables, frequencies and percentages were presented.

## 3 RESULTS

### 3.1 Patient and tumor characteristics

**FIGURE 2:**
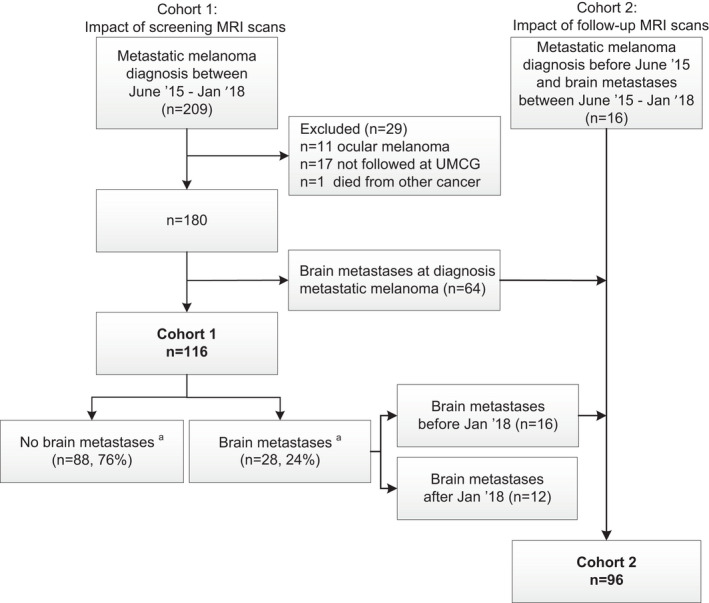
Consort diagram of the study cohorts. The impact of screening MRI scans was evaluated in patients diagnosed with metastatic melanoma without brain metastases between June 2015 and January 2018 (Cohort 1). The impact of follow‐up MRI scans was examined in patients diagnosed with brain metastases between June 2015 and January 2018 (Cohort 2)

In 116 patients, the impact of screening MRI scans was determined (Cohort 1, Figure 2). Median age at time of metastatic melanoma diagnosis was 66 years (range: 21–86), 60 (52%) were female, and 64 (55%) had a *BRAF*‐mutation. The median time between metastatic melanoma diagnosis and diagnosis of brain metastases or last follow‐up (death or end of study follow‐up) was 13.1 months (range: 0–24). Patient and tumor characteristics and anti‐tumor treatments received are presented in Table 1.

**TABLE 1:** Patient, tumor, and treatment characteristics

Variable	Impact of screening MRI scans *N* = 116; *N* (%)	Impact of follow‐up MRI scans *N* = 96; *N* (%)
Median age (range)^a^	66 (21–86)	63 (35–90)
Female	60 (52%)	41 (43%)
*BRAF*‐mutation present	64 (55%)	66 (69%)
LDH^a^		
<1 ULN	77 (66%)	61 (64%)
1–2.5 ULN	23 (20%)	22 (23%)
>2.5 ULN	8 (7%)	7 (7%)
Missing	8 (7%)	6 (6%)
Diagnosis of brain metastases <2 years after metastatic melanoma diagnosis	28 (24%)	93 (97%)
Median time of follow‐up, months (range)^b^	13.1 (0–24)	7.7 (0–12)
1‐year overall survival^c^	76 (66%)	42 (44%)
2‐year overall survival^c^	52 (45%)	19 (20%)
Systemic treatments^d^		
BRAF/MEK inhibitors	37 (32%)	54 (56%)
Immune checkpoint inhibitors	84 (72%)	56 (58%)
Chemotherapy	5 (4%)	3 (3%)
Localized treatments for brain metastases^d^		
SRT	–	34 (35%)
WBRT	–	15 (16%)
(Neuro)surgery	–	18 (19%)

^a^ At the time of diagnosis of metastatic melanoma or brain metastases.

^b^ Interval between diagnosis of metastatic melanoma to diagnosis of brain metastases or last follow‐up or diagnosis from brain metastases to last follow‐up.

^c^ From diagnosis of metastatic melanoma or brain metastases.

^d^ Received treatments within the first 2 years after the diagnosis of metastatic melanoma (until brain metastases diagnosis or end of follow‐up) and within the first year after the diagnosis of brain metastases. Patients could have received multiple systemic and localized treatments.

In 96 patients, the impact of 3‐monthly follow‐up MRI scans was determined (Cohort 2, Figure 2). The median age at time of brain metastases diagnosis was 63 years (range: 35–90), 41 (43%) were female, and 66 (69%) harbored a *BRAF*‐mutation (Table 1). The median time between brain metastases diagnosis and last follow‐up was 7.7 months (range: 0–12).

#### 3.1.1 Impact of screening MRI scans

In total, 238 MRI scans were performed in the first 2 years after metastatic melanoma diagnosis, of which 101 MRI scans (42%) were performed as baseline imaging. In 15 cases, no baseline MRI scan was performed. Reasons to omit baseline MRI scans were poor performance status (*n* = 6), wish for no further evaluation (*n* = 2), claustrophobia (*n* = 1), and in six patients the reason was unknown. Of the 238 MRI scans, 102 were screening MRI scans (43%). In total, 36% of the advised 6‐monthly screening MRI scans were performed (Table 2), and in 56 patients (48%) no screening MRI scans were performed. Of the 56 patients without screening MRI scans, 30 patients (54%) died or were diagnosed with brain metastases within 6 months after the diagnosis of metastatic melanoma. Other reasons to omit screening MRI scans were claustrophobia (*n* = 2) and no further treatment options (*n* = 3), and in the remaining 21 patients the reason to not perform screening MRI scans was unknown. Twenty‐five additional MRI scans (11%) were performed due to neurological symptoms. The remaining 11 MRI scans (5%) were performed for the follow‐up of a skull metastasis (*n* = 3), the evaluation of hypophysitis (*n* = 4), or extra response assessment (n=4). A swimmer plot including the performed MRI scans and diagnosis of brain metastases is available in Figure S1.

**TABLE 2:** Adherence to advised 6‐monthly screening and 3‐monthly follow‐up MRI scans

Screening MRI scans				
Time interval (months)^a^	No. of patients alive and without BM^b^	No. of patients with screening MRI scan^c^	Percentage of patients with screening MRI scan	Diagnosis of asymptomatic BM
0–6	79	18	23%	1
6–12	69	33	48%	5
12–18	60	25	42%	1
18–24	51	15	29%	1
			Average: 36%	Total: 8

Follow‐up MRI scans				
Time interval (months)^a^	No. of patients alive^b^	No. of patients with follow‐up MRI scan^d^	Percentage of patients with follow‐up MRI scan	Changes in treatment strategy
0–3	76	37	49%	22
3–6	60	43	72%	24
6–9	47	36	77%	20
9–12	42	31	74%	9
			Average: 68%	Total: 75

Abbreviations: BM, brain metastases; No., number.

^a^ Time from diagnosis of metastatic melanoma or melanoma brain metastases.

^b^ At upper limit of the scan interval

^c^ Six patients had screening MRI scans but were not alive at upper limit of scan interval and five patients had two screening MRI scans within 6 months.

^d^ Twelve patients had follow‐up MRI scans but were not alive at upper limit of scan interval and nine patients had two follow‐up MRI scans within 3 months.

Of the 116 patients, 28 patients (24%) developed brain metastases within the first 2 years after metastatic melanoma diagnosis (Figure 2). In the 60 patients in which at least one screening MRI scan was performed, 17 patients (28%) developed brain metastases. In 11 of those patients (65%), the brain metastases were detected by screening MRI scans, and in most cases there were no symptoms (8/11, 73%). The onset of neurological symptoms led to the diagnosis of brain metastases in six out of 17 (35%) patients. The diagnosis of asymptomatic brain metastases by the screening MRI scans resulted in treatment changes in all eight patients, and these changes included additional SRT (*n* = 4), change in systemic treatment (*n* = 1), and shortening of scan interval to less than 3 months (*n* = 3). In the 56 patients without screening MRI scans, 11 patients (20%) developed brain metastases and 8 out of these 11 patients (73%) were symptomatic. In the remaining three patients (27%), brain metastases were diagnosed due to a suspicious brain lesion on PET imaging (*n* = 1) and additional baseline MRI scans before first‐line treatment (*n* = 2).

#### 3.1.2 Impact of follow‐up MRI scans

In 96 patients, 352 MRI scans were performed, including 107 baseline MRI scans (30%), 168 follow‐up MRI scans (48%), 38 scans for localized treatments (11%), and 22 scans (6%) due to the occurrence of neurological symptoms (6%). The remaining 17 MRI scans (5%) were performed for other indications, including additional response assessment (*n* = 16) and the evaluation of an abscess after craniotomy (*n* = 1). In total, 68% of the planned 3‐monthly follow‐up MRI scans were performed (Table 2). A swimmer plot including the performed MRI scans and treatment strategy changes after follow‐up MRI scans is available in Figure S2.

### 3.2 Changes in treatment strategy

**FIGURE 3:**
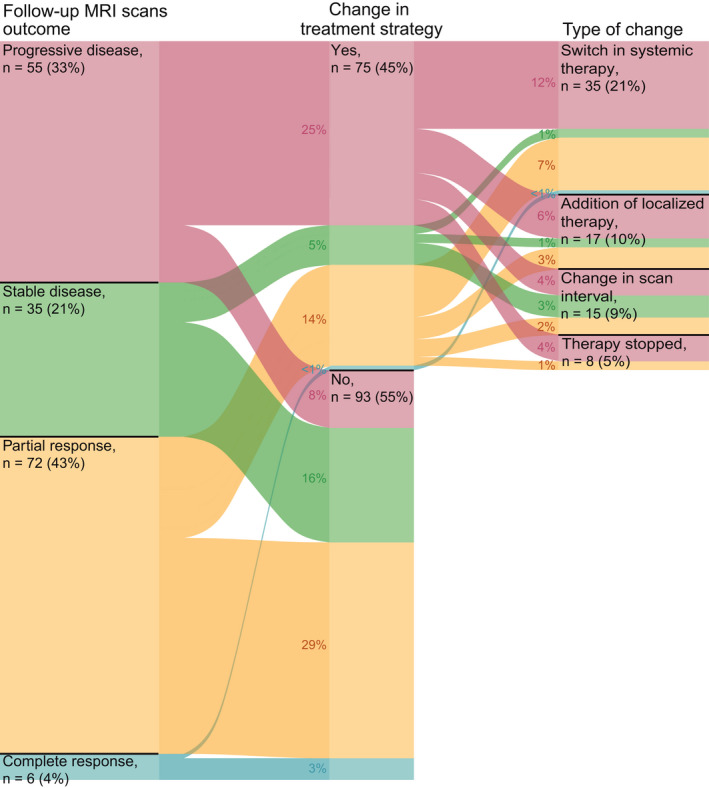
Alluvial plot for the proportion of follow‐up MRI scans after which treatment strategy was changed versus follow‐up MRI scans after which no treatment change was observed, stratified by MRI outcomes. Scans after which a change in treatment strategy was observed are further subdivided into type of treatment change

Changes in treatment strategy were observed after 75 out of 168 follow‐up MRI scans (45%, Figure 3). The scan outcome influenced the change in treatment in 67 out of 75 cases (89%). The treatment strategy changed in the remaining eight patients due to extracranial progression (*n* = 6) and treatment‐related toxicity (*n* = 2). In 42 cases, the change in treatment strategy was due to progressive disease and included changes in systemic treatment (*n* = 20), implementation of localized treatment (*n* = 9), shortening of scan interval (*n* = 7), and cessation of anti‐tumor treatment (*n* = 6). Changes after MRI scans showing stable disease (*n* = 9) were mainly shortening of scan intervals due to mixed treatment responses (*n* = 4). Of the 23 changes observed after MRI scans showing partial response, 12 included a change in systemic treatment. The change in systemic treatment mainly included a change from BRAF/MEK inhibitors to immune checkpoint inhibitors (10 out of 12).

### 3.3 Impact of follow‐up MRI scans in time on treatment

**FIGURE 4:**
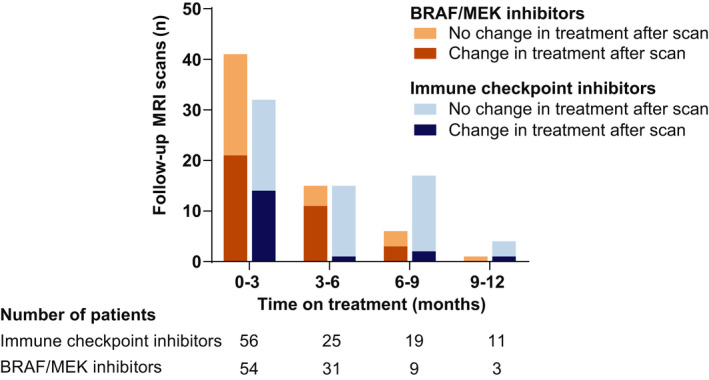
Bar plots showing the number of MRI scans after which a change in treatment strategy was observed stratified by type of systemic treatment receiving at time of scanning. Timeframes indicate duration of systemic treatment at time of scanning. Patients who received multiple systemic treatment lines were repeatedly included at the start of a new line of treatment

BRAF/MEK inhibitors were prescribed to 54 patients with brain metastases (56%, Figure 4), which is 80% of patients with a *BRAF*‐mutation (53 out of 66). Additionally, one symptomatic patient received BRAF/MEK inhibition for a few days while awaiting mutation analysis, which was eventually *BRAF* wild type. Of all the patients treated with BRAF/MEK inhibitors, nine patients (17%) were found to have a durable response (≥6 months). Within the first 6 months on treatment, treatment changes were observed after 32 out of 56 follow‐up MRI scans (57%). Of the 32 scans after which a change in treatment was observed, the scans showed progression (*n* = 13), stable disease (*n* = 3), partial response (*n* = 15), and a complete response (*n* = 1). Only seven MRI scans were performed in patients that responded to BRAF/MEK inhibitors for at least 6 months. After three of those seven scans (43%) the treatment strategy changed and all three scans showed progressive disease.

Immune checkpoint inhibitors were administered to 56 patients (58%, Figure 4), and durable responses were observed in 19 patients (34%). In the first 6 months, treatment changes were observed after 15 out of 47 follow‐up MRI scans (32%). Of those 15 follow‐up MRI scans whereafter the treatment changed, 11 showed progression, three stable disease, and one partial response. The number of follow‐up MRI scans resulting in a change in treatment strategy decreased in time on immune checkpoint inhibitors. From 6 months on treatment to the end of the first year after brain metastases diagnosis, treatment changes were observed after three out of 21 follow‐up MRI scans (14%). All three scans showed partial intracranial response, and the treatment changed due to oligo‐extracranial progression (*n* = 2) and treatment toxicity (*n* = 1).

## 4 DISCUSSION

In the current study, one third of the brain metastases, diagnosed within the first 2 years after the diagnosis of metastatic melanoma, were detected asymptomatically by 6‐monthly screening MRI scans. Furthermore, treatment strategy changes were observed after 45% of the 3‐monthly follow‐up MRI scans. Changes in treatment strategy after follow‐up MRI scans occurred less frequently in patients with durable responses to immune checkpoint inhibitors.

Early diagnosis of brain metastases has become increasingly relevant with the implementation of effective treatment options and better treatment outcomes in asymptomatic patients with small volume brain metastases.^13^, ^16^, ^18^ Nevertheless, clear guidelines on the use of screening MRI scans in patients with metastatic melanoma are lacking.^23^, ^24^, ^25^ Our study demonstrates the potential benefit of regular screening MRI scans in patients with metastatic melanoma in the current treatment era. In our study, almost a quarter of the patients diagnosed with metastatic melanoma, without brain metastases at diagnosis, developed brain metastases within 2 years after diagnosis. A recent, single‐center study without a defined imaging protocol, reported a cumulative brain metastases incidence of 52% (55 out of 106) in patients with metastatic melanoma.^4^ In 66 patients with metastatic melanoma without brain metastases at diagnosis, 15 patients (23%) developed brain metastases during the follow‐up period. This is comparable to the number of patients developing brain metastases in our cohort. In the patients who had at least one screening MRI scan in the current study, the majority of brain metastases were diagnosed asymptomatically by screening MRI scans. In contrast, in patients who lacked screening MRI scans, almost three quarters of the brain metastases were diagnosed symptomatically. However, over half of the patients without screening MRI scans died or were diagnosed with brain metastases within 6 months after metastatic melanoma diagnosis, which is before the first planned screening MRI scan. Moreover, for most other patients, the reason why the oncologist opted to omit screening MRI scans was unknown. Therefore, these data must be interpreted with caution.

Two other studies, performed prior to the availability of effective treatment options, examined the early diagnosis of melanoma brain metastases using regular brain imaging.^3^, ^28^ Asymptomatic brain metastases were detected by staging CT scans prior to the commencement of interleukin‐2 treatment in 12% of metastatic melanoma patients between 1995 and 2009.^28^ Furthermore, 39% of metastatic melanoma patients that were enrolled in clinical trials including 6‐weekly brain imaging (CT or MRI scans) between 1986 and 2004 were diagnosed with brain metastases.^3^ Our study results can guide the design of future prospective, randomized studies on the optimal scan interval and cost‐effectiveness of screening MRI scans. These subsequent studies may also identify relevant patient and disease factors influencing the optimal scan interval for the individual patient. Potential determinants relevant for the optimal screening MRI scan interval are melanoma location and type, metastatic sites, *BRAF*‐ and *NRAS*‐mutational status, and LDH‐level at diagnosis. Furthermore, extracranial and intracranial treatment responses and the occurrence of neurological symptoms need to inform decisions regarding brain imaging in patients with metastatic melanoma. Screening for asymptomatic brain metastases may also be relevant for patients with other tumor types with a high brain metastases incidence, such as lung and breast cancers, especially since effective treatment options are also emerging for intracranial disease in those tumor types.^29^, ^30^, ^31^

After 45% of the follow‐up MRI scans, a change in treatment strategy was observed. A previous study evaluated the use of follow‐up MRI scans in patients treated with SRT.^32^ Scans were performed 2, 4, 6 months after SRT and every 3 months after that. Using that imaging protocol, 62% of intracranial progression was detected at an asymptomatic stage. Nevertheless, they did not evaluate the impact of the MRI scans in patients without SRT or whether the follow‐up MRI scans led to treatment strategy changes. A subsequent study reported that early detection of asymptomatic intracranial progression using follow‐up brain imaging was cost‐effective due to less use of neurosurgical interventions and lower expenses for the management of neurological symptoms.^33^ The current study demonstrates that follow‐up MRI scans often result in changes in the treatment strategy of patients with melanoma brain metastases and are together with CT scans evaluating the extracranial disease status of great importance for the management of patients with metastatic melanoma and brain metastases.

We frequently observed treatment strategy changes after follow‐up MRI scans in patients treated with BRAF/MEK inhibitors. This may be clarified by proactively switching from BRAF/MEK inhibition to immune checkpoint inhibitors in responding patients in selected cases, next to the limited duration of intracranial response to BRAF/MEK inhibition.^11^, ^13^, ^14^ In patients receiving immune checkpoint inhibitors, the number of treatment changes after follow‐up MRI scans decreased in time on treatment. The latter may be explained by the higher frequency of treatment failures occurring in the first 6 months of immune checkpoint inhibitor therapy compared to the occurrence of treatment failure beyond 6 months of therapy.^6^, ^7^, ^34^, ^35^ In our study, the changes in treatment strategy within the first 6 months on immune checkpoint inhibitors were mostly observed after scans showing progression. In contrast, after 6 months on treatment, the treatment changed due to other reasons than intracranial progression. It might be hypothesized that the scan interval can be prolonged in patients with a durable response to immune checkpoint inhibitors, while continuing 3‐monthly follow‐up MRI scans in patients on BRAF/MEK inhibitors may be more appropriate to monitor for disease progression or a “therapeutic window” to commence immune checkpoint inhibitors, when clinically possible.

The retrospective nature of this study is a limitation. Patients were retrospectively selected from a registry to determine the impact of screening and follow‐up MRI scans. In those patients, 6‐monthly screening and 3‐monthly follow‐up MRI scans were performed in 36% and 68% of patients, respectively. The charts often lacked documentation of reasons for omitting the scans. Prospective, randomized studies with stricter scan interval protocols are needed to determine the optimal scan interval of screening and follow‐up MRI scans and the impact of regular brain imaging on survival of patients with metastatic melanoma with and without brain metastases. Furthermore, reasons to change treatment were not systemically recorded. Therefore, two medical melanoma oncologists independently determined the influence of follow‐up MRI scans on treatment changes retrospectively. The current study was a single‐center study. Therefore, the oncologists could have also been the treating physician. This may have influenced the assessment of the contribution of the scan outcomes.

In conclusion, screening MRI scans in patients with metastatic melanoma aid in the early detection of brain metastases before neurological symptoms occur. Furthermore, regular follow‐up MRI scans in patients with melanoma brain metastases lead to changes in treatment strategy. In patients with durable response to immune checkpoint inhibitors, the changes in treatment strategy after follow‐up MRI scans decreased in time on treatment. More research is warranted to determine the impact and cost‐effectiveness of regular brain imaging and subsequent treatment changes in survival and to determine the optimal scan interval of screening and follow‐up MRI scans.

## CONFLICT OF INTEREST

Geke A.P. Hospers is a consultant/advisory board member for Amgen, Roche, Merck, Bristol Myers Squibb, Pfizer, Novartis, and Pierre Fabre, and has received grants from Bristol Myers Squibb and Seerave, outside the submitted work and paid to the institution. Mathilde Jalving has served as an advisory board member for Bristol Myers Squibb, Novartis, Merck, and Pierre Fabre, fees paid to the institution. The other authors have no conflict of interest to disclose.

## ETHICS STATEMENT

The UMCG review board granted ethical approval and waived the need for an informed consent procedure (METc2017/511 and METc2019/361). The “opt‐out” register was assessed to exclude patients who disapprove of routinely collected data being used for research purposes.

## Supporting information

Supplementary Material

## Data Availability

The datasets used and/or analyzed during the current study are available upon reasonable request.
