# Supplementary material for: Value of screening and follow‐up brain MRI scans in patients with metastatic melanoma
Source: Cancer Med. 2021 Nov 5;10(23):8395–404. doi: 10.1002/cam4.4342 (PMC8633235; doi:10.1002/cam4.4342)
Supplement: Supplementary file 1 — Supplementary Material [file CAM4-10-8395-s001.docx]

**Supporting information**

**Figure 1** Swimmer plot of patients diagnosed with metastatic melanoma and MRI scans performed within the first two years after diagnosis of metastatic melanoma Abbreviation BM: brain metastases.

**Figure 2** Swimmer plot of patients diagnosed with melanoma brain metastases and MRI scan performed within the first year after diagnosis of melanoma brain metastases.
